# Genome-wide variation in cell-free DNA end-motif entropy predicts immunotherapy response in head and neck cancer

**DOI:** 10.1172/JCI196284

**Published:** 2026-05-19

**Authors:** Ravi Bandaru, Hailu Fu, Haizi Zheng, Jocelyn Liang, Li Wang, Shuchi Gulati, Benjamin H. Hinrichs, Mingxiang Teng, Bin Zhang, Masha Kocherginsky, De-Chen Lin, David A. Hildeman, Francis P. Worden, Matthew Old, Neal E. Dunlap, John M. Kaczmar, Maura L. Gillison, Dalia El-Gamal, Trisha Wise Draper, Yaping Liu

**Affiliations:** 1 Department of Biochemistry and Molecular Genetics, Feinberg School of Medicine, Northwestern University, Chicago, Illinois, USA.; 2 Robert H. Lurie Comprehensive Cancer Center of Northwestern University, Chicago, Illinois, USA.; 3 Division of Human Genetics, Cincinnati Children’s Hospital Medical Center, Cincinnati, Ohio, USA.; 4 Division of Hematology/Oncology, and; 5 Department of Pathology, University of Cincinnati, Cincinnati, Ohio, USA.; 6 Department of Biostatistics and Bioinformatics, Moffitt Cancer Center, Tampa, Florida, USA.; 7 Department of Pediatrics, University of Cincinnati College of Medicine, Cincinnati, Ohio, USA.; 8 Division of Biostatistics and Epidemiology, Cincinnati Children’s Hospital Medical Center, Ohio, USA.; 9 Department of Preventive Medicine, Feinberg School of Medicine, Northwestern University, Chicago, Illinois, USA.; 10 Center for Craniofacial Molecular Biology, Herman Ostrow School of Dentistry, and Norris Comprehensive Cancer Center, University of Southern California, Los Angeles, California, USA.; 11 Division of Immunobiology, University of Cincinnati College of Medicine, Cincinnati, Ohio, USA.; 12 University of Michigan Cancer Center, Ann Arbor, Michigan, USA.; 13 Department of Otolaryngology, Ohio State University, Columbus, Ohio, USA.; 14 Department of Radiation Oncology, University of Louisville, Louisville, Kentucky, USA.; 15 Division of Hematology/Oncology, Medical University of South Carolina, Charleston, South Carolina, USA.; 16 The University of Texas MD Anderson Cancer Center, Houston, Texas, USA.

**Keywords:** Genetics, Oncology, Cancer immunotherapy, Epigenetics, Head and neck cancer

## Abstract

**BACKGROUND:**

Minimally invasive biomarkers predicting the immunotherapy response in head and neck squamous cell carcinoma (HNSCC) remain an unmet clinical need.

**METHODS:**

In a prospective, multi-institutional phase II trial, we performed whole-genome sequencing of 185 longitudinal plasma cell-free DNA (cfDNA) samples from 68 patients with locally advanced, surgically resectable HNSCC who received neoadjuvant and adjuvant pembrolizumab. We developed the regional motif diversity score (rMDS), a fragmentomic metric that quantifies the entropy of cfDNA 5′-end motifs across genomic regions.

**RESULTS:**

Unsupervised analysis showed that rMDS robustly distinguished responders from nonresponders, outperforming established fragmentomic metrics and copy number alterations while remaining independent of technical confounders. Longitudinal rMDS changes localized to regions enriched for immune-, lectin-, and keratinization-related genes — hallmarks of squamous cell carcinoma — reflecting tumor–peripheral immunity interplay during treatment. The most dynamic regions clustered at telomere-proximal loci, suggesting a link between telomere biology and cfDNA fragmentation. An rMDS-based machine learning classifier achieved AUC 0.89–0.99 across validation settings, with the highest accuracy after treatment, outperforming PD-L1 expression and tumor fraction in matched samples. Predicted responders showed improved disease-free survival (log-rank *P* = 0.035; HR 2.67, 95% CI 1.03–6.92).

**CONCLUSION:**

rMDS represents a biologically meaningful and clinically actionable biomarker for the immunotherapy response in HNSCC, and merits integration into future risk assessment frameworks.

**TRIAL REGISTRATION:**

ClinicalTrials.gov NCT02641093.

**FUNDING:**

National Human Genome Research Institute (NHGRI), NIH grant R56HG012360; startup funds from Cincinnati Children’s Hospital Medical Center, Northwestern University, and Robert H. Lurie Comprehensive Cancer Center; Science Olympiad Alumni Research Grant, Science Olympiad USA Foundation; Merck Sharp & Dohme Corp.

## Abstract 2

A simple blood test may help predict which head and neck cancer patients will benefit from immunotherapy, enabling more personalized treatment decisions.

## Introduction

Head and neck squamous cell carcinoma (HNSCC) is the seventh most prevalent cancer globally and is characterized by high recurrence (~50%) and poor survival rates despite aggressive multimodal treatment strategies (1–3). Immune checkpoint blockers, particularly those targeting programmed death (ligand) 1 (PD-1/PD-L1), such as pembrolizumab and nivolumab, have demonstrated promising clinical outcomes and significant benefit to select patient groups (4, 5). However, primary resistance remains prevalent, with only approximately 18% of patients with HNSCC achieving substantial responses to immunotherapy (5, 6). These findings highlight an urgent need for reliable biomarkers capable of predicting immunotherapy responses to facilitate personalized therapeutic strategies. Current biomarkers, including PD-L1 expression, tumor mutational burden, and immune gene expression profiles, have been extensively investigated but demonstrate limited sensitivity and specificity in differentiating responders from nonresponders and frequently require invasive tissue biopsies (7).

Fragmentation patterns of circulating cell-free DNA (cfDNA) in plasma have recently emerged as a promising class of minimally invasive biomarkers for predicting and monitoring therapeutic responses in cancer, particularly in the context of immunotherapy (8–10). cfDNA, which is released into the bloodstream through cellular apoptosis, necrosis, and active secretion, carries a wealth of information reflecting the genomic and epigenomic landscape of its cells of origin (11–16). Recent advances in sequencing technologies and bioinformatics have enabled detailed analysis of cfDNA fragmentation patterns, such as fragment size distributions, genome-wide coverage, DNA evaluation of fragments for early interception (DELFI) (17), and the sequence context of fragment ends (18). In particular, end motifs of cfDNA fragments and derived metrics like the motif diversity score (MDS) have emerged as robust fragmentomic biomarkers (18). For example, characteristic motif alterations at cfDNA fragment ends and elevated MDSs have been consistently observed in HNSCC and other malignancies (18). These fragmentomic features have demonstrated the potential to detect the presence of cancer, infer the tissue of origin, and track disease progression with high sensitivity and specificity (11, 12).

Building on these advances, several studies have sought to explore the clinical utility of cfDNA fragmentation patterns as biomarkers for immunotherapy responses in patients with cancer (8, 9). However, these investigations have been retrospective and single-institution efforts. cfDNA fragmentation was analyzed primarily as a supplemental metric to enhance the detection of copy number alterations, rather than as a standalone biomarker. Moreover, these studies only included a small number of patients per cancer type or pooled samples across diverse malignancies, limiting the generalizability and interpretability of their findings. Notably, to our knowledge, no prospective studies have yet systematically evaluated cfDNA fragmentation features as predictive or prognostic biomarkers for cancer immunotherapy across multiple institutes, especially in patients with HNSCC.

In a recent multi-institutional prospective phase II clinical trial (NCT02641093) (19), we investigated the efficacy of neoadjuvant and adjuvant pembrolizumab in patients with surgically resectable, locally advanced HNSCC (19). The trial demonstrated improved 1-year disease-free survival (DFS) in intermediate-risk patients compared with historical controls. These findings were recently validated by the phase III KEYNOTE-689 (NCT03765918), which showed improved DFS with neoadjuvant and adjuvant pembrolizumab and established this approach as a new standard of care for locally advanced HNSCC (20). Nonetheless, conventional predictive biomarkers, including PD-L1 expression and immune gene signatures, proved inadequate in accurately stratifying patient responses, highlighting the limitations of existing molecular predictors. Motivated by these findings, we explored whether cfDNA fragmentation patterns could predict response to pembrolizumab immunotherapy in patients enrolled in this clinical trial. We conducted longitudinal analyses using low-coverage (~1.9×) cfDNA whole-genome sequencing (WGS) of plasma samples collected at 3 different time points before and after treatment, ensuring balanced demographic characteristics between the responder and nonresponder groups. We developed a fragmentomic metric, termed the regional motif diversity score (rMDS), designed to quantify genome-wide variations in the entropy of end motifs across distinct genomic regions. Remarkably, unsupervised analysis using rMDS effectively distinguished immunotherapy responders from nonresponders and outperformed conventional fragmentomic features and other biomarkers from the same patient cohort. This approach captures changes in cfDNA fragmentation undetectable by traditional biomarkers, thereby providing a noninvasive method for response monitoring and enhancing clinical risk stratification.

## Results

### Generation of cfDNA WGS from longitudinal plasma samples in a multi-institutional phase II trial.

**Figure 1:**
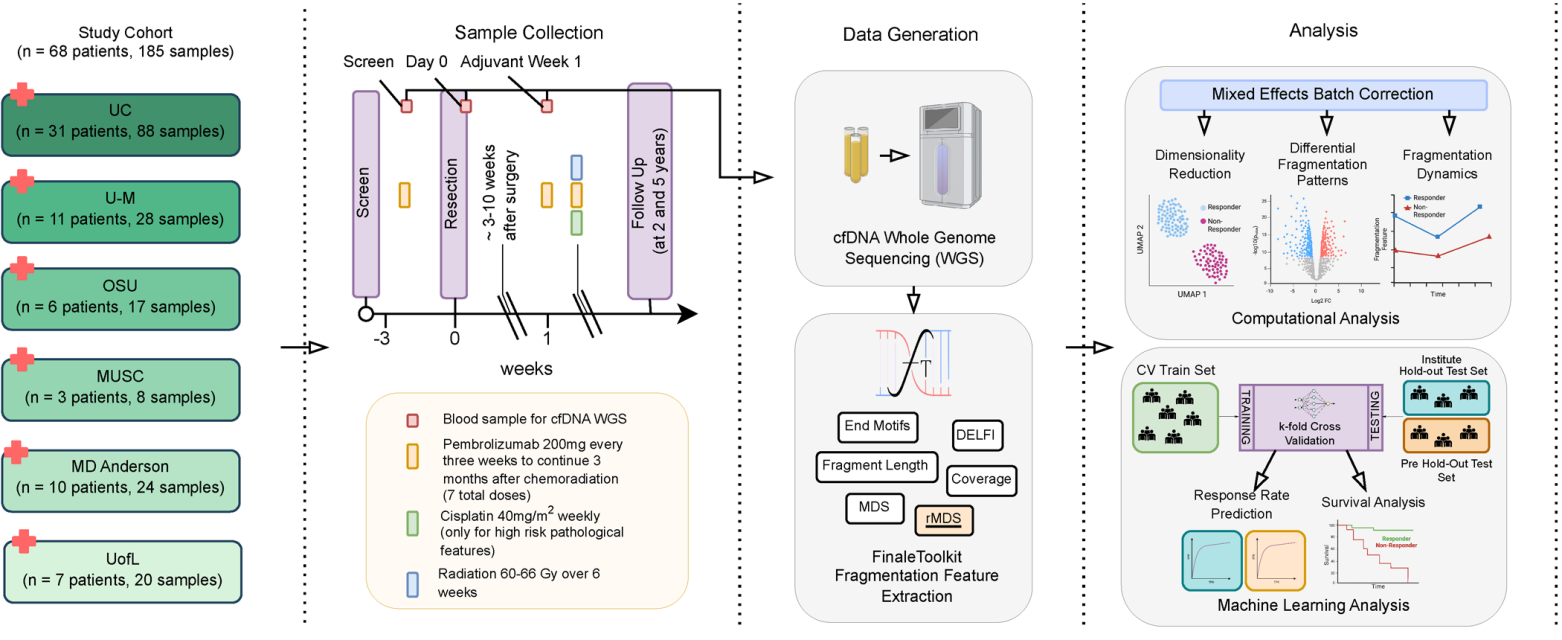
Experimental design and analytical workflow for the HNSCC cohort. Patients with head and HNSCC underwent surgical tumor resection and pembrolizumab immunotherapy, followed by adjuvant radiation therapy (60–66 Gy over 6 weeks) and cisplatin (40 mg/m^2^) for high-risk pathological features. A total of 185 blood samples were collected at defined intervals throughout the course of treatment and follow-up from 68 patients across 6 institutions (UC, U-M, OSU, MUSC, MDACC, and UofL). Plasma-derived circulating cfDNA was isolated from blood samples collected at 3 different time points (screen, day 0, and adjuvant week 1) and subjected to WGS. Fragmentation features were extracted using FinaleToolkit, including MDS, single genome-wide summary score), rMDS, end-motif frequencies, DELFI, genome-wide coverage, and fragment length distributions. CV, cross-validation.

Our cohort comprised 68 patients from the original cohort of 92 patients with locally advanced, surgically resectable HNSCC who had available plasma samples and were enrolled in a prospective, multi-institutional phase II clinical trial. For a subset of patients in the original cohort, only whole blood was collected, and these samples were excluded because genomic DNA contamination precludes accurate cfDNA fragmentation profiling. Thus, the current cohort spanned 6 academic institutes and included 40 clinical full or partial responders and 28 nonresponders, as determined by treatment effect (TE) (see Methods), with no significant differences in demographic or clinical variables between the response groups (Table 1, Figure 1, and Supplemental Table 1; supplemental material available online with this article; https://doi.org/10.1172/JCI196284DS1). All patients received a single 200 mg i.v. dose of neoadjuvant pembrolizumab, followed by surgical resection and adjuvant radiotherapy (60–66 Gy). High-risk patients received concurrent weekly cisplatin (40 mg/m^2^), and all patients were eligible to receive postoperative pembrolizumab for up to 7 total doses. Plasma (0.5 mL) was collected at 3 defined clinical time points: prior to neoadjuvant therapy (screen), the day of surgery (day 0), and 3–10 weeks after surgery (adjuvant week 1).

Of the 185 cfDNA WGS libraries generated from plasma samples, 176 met predefined quality control criteria (details are provided in Methods), achieving a median sequencing coverage of approximately 1.9× per sample (Supplemental Figure 1A and Supplemental Table 2). From these, 10 patients (i.e., 25 samples) were randomly set aside as a prespecified holdout set for the later evaluation of the machine learning model, and the analysts remained blinded to these samples. The remaining 151 samples from 58 patients constituted the analysis dataset used for all subsequent analyses, including unsupervised rMDS clustering, differential rMDS analysis, and machine learning model training and cross-validation (Supplemental Figure 1B). The pre-holdout set of 25 samples from 10 patients was reserved exclusively for the evaluation of model generalizability and was not used in any upstream analyses. Forty-one patients provided samples across all 3 longitudinal time points, with sample distribution approximately balanced among visits (Supplemental Figure 2A). Samples from the University of Cincinnati (UC) constituted a greater proportion (48%) compared with other institutions (Supplemental Figure 2B). Responders and nonresponders were balanced in the analysis set. Technical metadata, including cfDNA isolation and library preparation dates, showed no systematic biases or imbalances between the analysis set and the pre-holdout set (Supplemental Figure 2, C and D).

### Unsupervised analysis of rMDS distinguishes immunotherapy responders from nonresponders.

We first examined whether differences in fragmentation patterns existed between responders and nonresponders. Given the low sequencing coverage, we partitioned the reference genome into nonoverlapping bins of 500 kb. Within each genomic bin, we utilized FinaleToolkit (21) to extract standard fragmentomic features, including fragment length, fragment coverage corrected for guanine + cytosine% (G+C%) content bias, and DELFI. Additionally, FinaleToolkit was used to compute genome-wide summary statistics for each sample, including frequencies of 5′ end motifs (*n* = 256) and MDS (*n* = 1) (18). Copy number variations (CNVs) for each genomic bin and tumor fractions for each sample were calculated using ichorCNA (22). To isolate the response-associated variance, we removed potential batch effects arising from plasma collection sites, cfDNA isolation procedures, and library preparation dates (details are provided in Methods; Supplemental Figure 3). Despite these analyses, none of these features demonstrated the separations between responders and nonresponders (silhouette scores 0.015–0.077; Supplemental Figure 4 and Supplemental Table 3).

**Figure 2:**
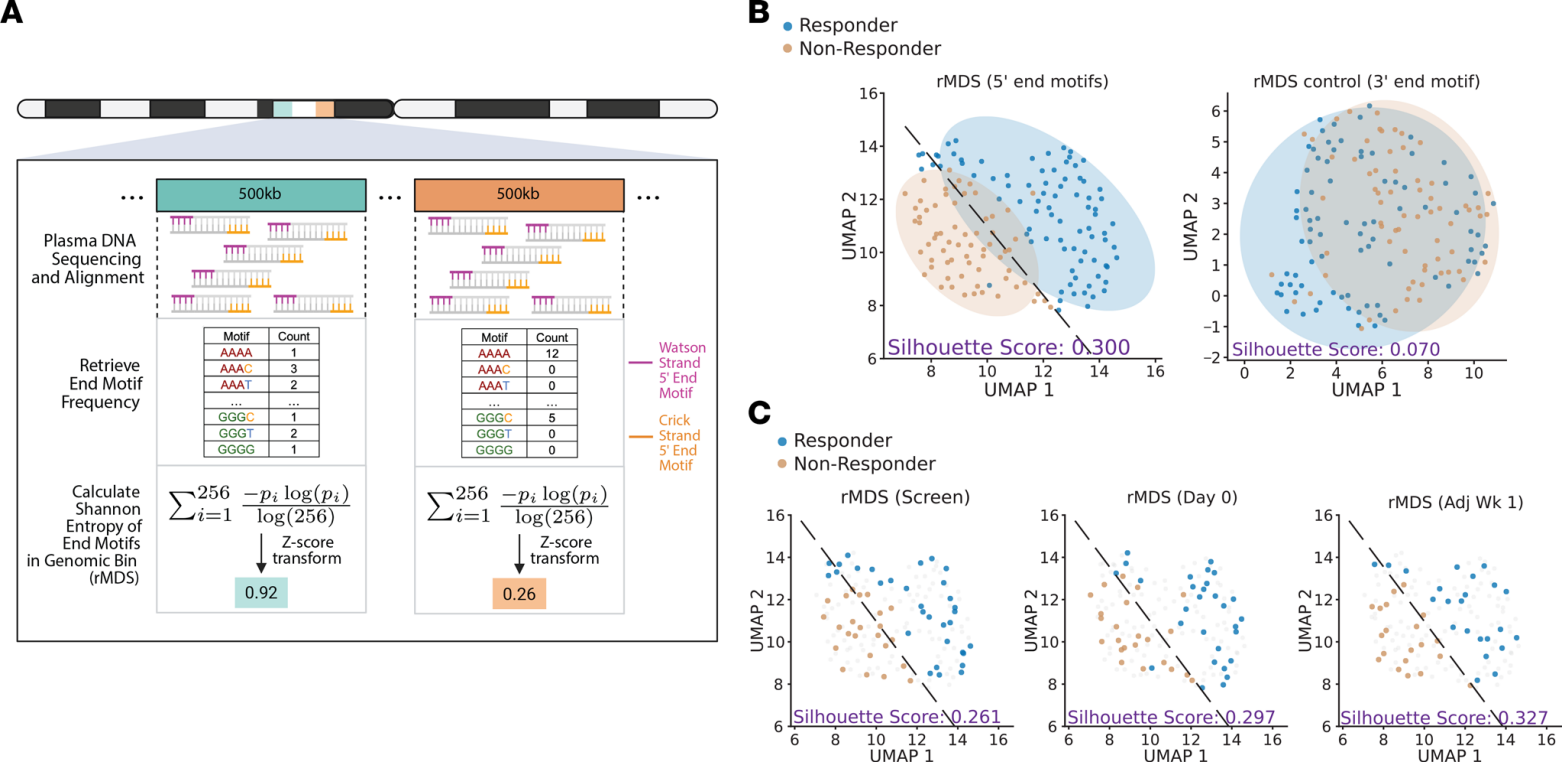
rMDS distinguishes immunotherapy responders from nonresponders. (**A**) Schematic representation of rMDS calculation: cfDNA 5′ end motifs are quantified within 500 kb genomic bins, and Shannon entropy is computed to yield rMDS, preserving regional information relative to genome-wide MDS. (**B**) UMAP visualization demonstrates clear separation of responders and nonresponders based on rMDS from 5′ end motifs that are related to cfDNA fragmentation, with minimal separation observed using 3′ end motifs as the control. (**C**) rMDS (5′ end motifs) profiles at screening, day 0, and adjuvant week 1 also independently separate responders from nonresponders. Silhouette scores quantify cluster separation. Ellipses represent approximately 2 SDs from group centroids.

A recent study has reported differences in genome-wide MDSs between patients with HNSCC and healthy individuals (18). However, the genome-wide MDS represents only a single aggregated metric of the variations in cfDNA 5′ end motifs. Given that the 5′ end of cfDNA fragments strongly correlates with cellular epigenetic profiles (18, 23), which vary substantially across genomic regions and reflect underlying gene-regulatory mechanisms (24, 25), evaluating end-motif diversity variations at a regional level across the genome could better capture this biological complexity. To address this, we developed a fragmentomic metric, termed the rMDS, which extends the genome-wide MDS by quantifying variations in the Shannon entropy of 5′ end 4 mer motifs across distinct genomic regions (Figure 2A). Remarkably, after correcting for batch effects, the *z* score transformed rMDS value effectively and unbiasedly discriminated immunotherapy responders from nonresponders in our cohort (Figure 2B). Interestingly, rMDS could differentiate between these 2 groups even at the pretreatment screening time point, suggesting that baseline differences in cellular epigenetic states may predict responsiveness to immunotherapy (Figure 2C). The greatest separation was observed at the most recent sample collection time point (adjuvant week 1, silhouette score = 0.327) compared with day 0 (silhouette score = 0.297) and screening (silhouette score = 0.261).

It is possible that the observed separation between the 2 groups could be attributed to potential batch effects specifically related to nucleotide sequence context or confounding changes in the overall nucleotide composition of cfDNA fragments, rather than genuine fragmentation patterns at the 5′ end. To address these concerns, we calculated the rMDS control based on 3′ end 4 mer motifs, which are not reported to be related to cfDNA fragmentation by the standard WGS approach we used. As expected, we found no differences in the 3′ end rMDS control between responders and nonresponders (silhouette score = 0.07), suggesting that the observed group separation by the 5′ end rMDS was not confounded by sequence composition or other technical effects (Figure 2B).

To assess the effect of sequencing depth on rMDS estimation, we performed empirical downsampling using the deeply sequenced BH01 cfDNA dataset from Snyder et al. (13) (~56× effective coverage). Reads were downsampled to 50×, 25×, 10×, 5×, 2×, 1×, and 0.1× coverage, and 500 kb rMDS profiles were recalculated (Supplemental Figure 6). Correlation with the full-depth reference increased monotonically with coverage, reaching *r* = 0.93 at 5× and remaining high at approximately 2× (*r* = 0.82), comparable to our study (~1.9×). Substantial decreases in correlation with the full-depth reference occurred only at extreme downsampling (0.1×). Downsampling of patient samples similarly demonstrated preserved responder/nonresponder discrimination at moderate reductions in coverage (silhouette scores = 0.3–0.42), with deterioration only at very low depth (silhouette score = 0.151; Supplemental Figure 7). These results suggest that, while deeper sequencing improved precision, a biologically meaningful rMDS structure was retained at the coverage used in our study.

We further evaluated the optimal k mer choice (Supplemental Figure 8). The 4 mer model demonstrated the strongest group separation (silhouette score = 0.3). Shorter 3 mers probably lacked sufficient sequence complexity, whereas 5 and 6 mers greatly expanded the feature space (1,024–4,096 different end motifs), resulting in sparse counts per end-motif type in each genomic bin at our sequencing depth (~1–4 fragments per end-motif type in each bin) and, thus, unstable entropy estimation. Therefore, 4 mers provided an optimal balance between contextual information and statistical robustness under the current low-coverage conditions.

### Longitudinal dynamics of differential rMDS patterns reveal telomere-associated fragmentation shifts.

**Figure 3:**
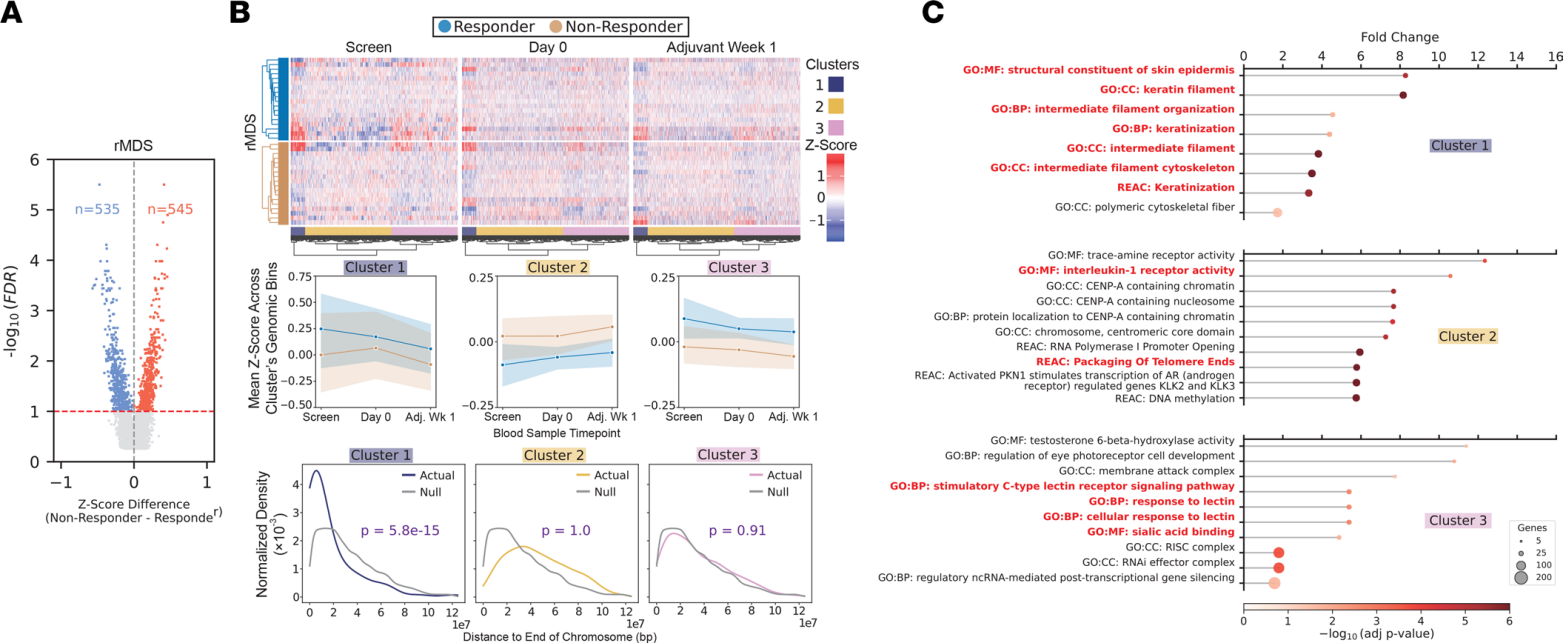
Differential rMDS regions between immunotherapy responders and nonresponders. (**A**) Volcano plot of differential rMDS genomic bins between responders and nonresponders across all samples. Regions with significantly increased or decreased motif diversity in responders are highlighted (linear mixed-effects model with empirical Bayes moderation; FDR <0.1). (**B**) Heatmap of differentially enriched rMDS regions across 3 time points (screen, day 0, adjuvant [Adj] week 1) reveals distinct longitudinal dynamics between responders and nonresponders, stratified by cluster. Middle panel: Line plots show the mean *z* score trajectory over time for each cluster, separated by response status. Bottom panel: Normalized density of distances from each rMDS region to the nearest chromosome end for each cluster, compared with a null distribution. *P* values indicate 1-sided Mann-Whitney *U* tests assessing whether the actual distribution was significantly closer to chromosome ends than the null. (**C**) GO enrichment analysis of differential rMDS clusters. Enrichment was tested using g:Profiler (hypergeometric test with FDR correction). The top 10 terms by fold enrichment are shown.

To identify genomic regions with differential rMDS patterns, we conducted a genome-wide comparative analysis of rMDS between immunotherapy responders and nonresponders. Besides the technical batch effect, other clinical variables, such as age and sex, could also affect the cfDNA fragmentation status. We further used a linear mixed-effects modeling approach to account for repeated measures across time points and different clinical covariates, including age, race, sex, ethnicity, diagnosis, smoking status, and alcohol use (details in Methods). After the correction for multiple hypothesis testing, we identified 1,080 significantly differential 500 kb bins (*q* < 0.1, Supplemental Table 4), including 545 genomic bins showing higher rMDSs in nonresponders and 535 bins showing lower rMDSs (Figure 3A). To explore the longitudinal dynamics of these differential rMDS regions, we applied joint hierarchical clustering to *z* score–transformed rMDS values measured across 3 longitudinal time points in the same patients (Figure 3B).

This clustering approach yielded 3 distinct clusters, each characterized by unique dynamic patterns of the rMDS. Cluster 1 showed the most pronounced divergence in rMDSs between responders and nonresponders, with initially large differences at screening that narrowed at day 0 before separating again by adjuvant week 1. Cluster 2 consistently showed higher rMDSs in nonresponders across all time points, whereas cluster 3 demonstrated the opposite pattern (Figure 3B).

Because the differential regions were defined using a statistical model that jointly evaluated response-associated differences across all time points while adjusting for patient-level covariates, we further examined the trajectories using covariate-corrected rMDS values. Consistent with the model results, visualization of covariate-adjusted signals confirmed substantial differences between responders and nonresponders for all 3 clusters (Supplemental Figure 9), supporting the robustness of the identified differential regions. These analyses clarify that the regions identified by the mixed-effects model captured longitudinal response-associated effects that may not have been fully apparent when visualizing unadjusted, only technically corrected, rMDS values alone.

Intrigued by the genomic locations of these clusters, we performed enrichment analysis. Remarkably, cluster 1 regions were significantly enriched near telomeric regions (Mann-Whitney *P* = 5.8 × 10^–15^, permutation *P* < 1 × 10^–5^), a feature absent in clusters 2 and 3 (Figure 3B). These findings suggest that distinct fragmentation dynamics near telomeric chromatin may be associated with the immunotherapy response.

### Gene ontology analysis links longitudinal dynamics of differential rMDS patterns to immune, lectin signaling, and keratinization programs.

Next, we sought to characterize the genes enriched in differential rMDS regions exhibiting distinct longitudinal dynamics. Gene ontology (GO) analysis identified unique functional signatures across rMDS clusters related to both immune system components and HNSCC tumor biology (Figure 3C and Supplemental Table 5). Specifically, cluster 1 was characterized by enrichment in keratinization, a defining histologic hallmark of squamous cell carcinomas, which constitute the predominant form of head and neck cancers. Particularly, the extent and type of keratinization were known to have prognostic value and were linked to differential survival outcomes (26–32). Cluster 2 was predominantly enriched for IL-1 and centromere protein A–related (CENPA-related) terms. Previous studies indicate that IL-1 signaling has been related to tumor growth and metastasis in HNSCC (33). Moreover, subnuclear patterns of CENPA have been shown to be correlated with immunotherapy responses in other cancer types (34) and chemotherapy disease curability in patients with HNSCC (35). Cluster 3 was characterized by enrichment in lectin signaling; previous studies have shown that genes within this group, such as *KLRK1*, are highly expressed in HNSCC and are linked to favorable prognosis and increased immune cell infiltration (36).

Representative rMDS *z* score trajectories at selected genomic loci further illustrated these patterns. A cluster 3 region located at chr12:52.0–52.5 Mb, encompassing keratin genes (e.g., *KRT7*, *KRT86*, and *KRT85*), showed an increase in rMDS in responders and a decrease in rMDS in nonresponders (Supplemental Figure 10). Another telomeric cluster 1 region at chr9:135.5–137.5 Mb demonstrated similar diverging trends (Supplemental Figure 10). Furthermore, loci enriched for CENPA-related and lectin signaling–related genes from clusters 2 and 3 displayed distinct group-specific fragmentation dynamics (Supplemental Figure 11).

### rMDS-based predictive modeling robustly discriminates immunotherapy responders across multiple validation scenarios.

**Figure 4:**
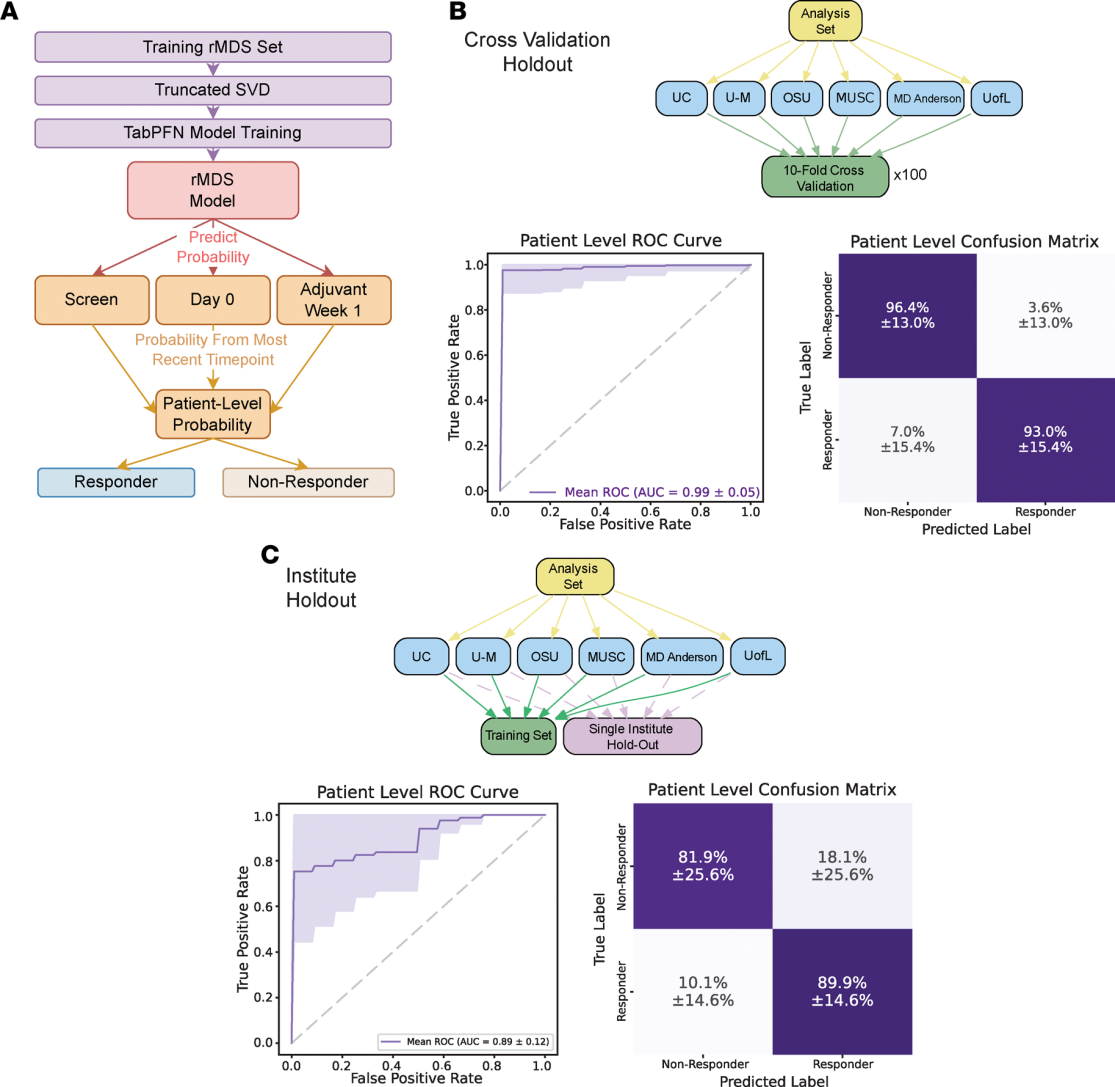
Predicting immunotherapy responses in HNSCC using rMDS. (**A**) Overview of the rMDS-based machine learning pipeline. Dimensionality reduction was first applied using truncated SVD. Dimensionality reduction was performed by truncated SVD, and reduced features were used to train TabPFN models. Patient-level probabilities were obtained by using the most recent available time point, then binarized as responder or nonresponder. (**B**) Cross-validation strategy. Models were trained using a 10-fold patient-level cross-validation repeated 100 times. (**C**) Institute holdout evaluation strategy. For each iteration, one institute was held out entirely for testing while the model was trained on the remaining institutions. The average ROC curve (mean ± SD) and confusion matrix (mean ± SD) are shown.

We next explored the potential of rMDS as a composite biomarker for predicting immunotherapy responses in patients with HNSCC. To mitigate overfitting due to limited sample sizes, we applied singular value decomposition (SVD) to genome-wide rMDS values, reducing feature dimensionality. Additionally, we utilized the Tabular Prior-data Fitted Network (TabPFN) classifier, which is specifically designed for machine learning studies with small sample sizes (37). Patient-level predictions were obtained using the most recent available time point, since our unsupervised analysis suggested greater rMDS separation at more recent time points (Figure 2C and Figure 4A).

We implemented a comprehensive 3-tier validation strategy to rigorously evaluate model performance under different scenarios. First, we assessed model accuracy through 10-fold patient-level cross-validation repeated 100 times on the analysis set. This approach yielded exceptional performance with a mean AUC of 0.99 ± 0.05 (Figure 4B). At 95% specificity, the model achieved 97.6% ± 10.1% sensitivity. Classification accuracy was consistently high across response categories, correctly identifying nonresponders 96.4% ± 13.0% of the time and responders 93.0% ± 15.4% of the time (Figure 4B). Sample-level analysis confirmed robust performance across all collection time points, with the highest AUC observed at adjuvant week 1 (0.99 ± 0.04), followed by screen (0.97 ± 0.09) and day 0 (0.95 ± 0.12) (Supplemental Figure 12 and Supplemental Table 6).

To evaluate real-world applicability in which samples originate from previously unseen institutions, we performed single-institute holdout validation within the analysis set (leave one institute out [LOIO]). Each institution was sequentially held out while training on samples from all other sites. This more stringent evaluation yielded a mean AUC of 0.89 ± 0.12 (Figure 4C), with a sensitivity of 75.3% ± 30.9% at 95% specificity. Classification accuracy remained robust, with correct identification of nonresponders and responders 81.9% ± 25.6% and 89.9% ± 14.6% of the time, respectively (Figure 4C). Consistent with previous analyses, adjuvant week 1 samples showed the highest discriminatory power (AUC = 0.94 ± 0.14), followed by screen (0.83 ± 0.15) and day 0 (0.82 ± 0.21) (Supplemental Figure 13 and Supplemental Table 7).

We assessed model generalizability by simulating the scenario of applying our predictor to entirely independent patient cohorts when the batch effects were not fully corrected. This pre-holdout analysis was included specifically to illustrate the effect of uncorrected technical variation on the predictive model. We randomly selected 10 patients (and their associated longitudinal samples) as a “pre-holdout” set across 100 iterations. This most stringent evaluation scenario yielded a mean AUC of 0.69 ± 0.19 (Supplemental Figure 14), with a sensitivity of 37.9% ± 27.0% at 95% specificity. While classification accuracy was more modest under these conditions, correctly identifying nonresponders 57.2% ± 26.5% of the time and responders 68.5% ± 20.0% of the time, performance remained above chance levels across all time points (AUC range: 0.67–0.68) (Supplemental Figure 15 and Supplemental Table 6). Together, these results demonstrate that when technical batch effects are properly accounted for, rMDS-based prediction models maintain robust performance under multiple validation conditions.

To further quantify the effect of batch correction on predictive performance, we directly compared model accuracy before and after correction in both cross-validation and institute holdout settings (Supplemental Figure 16). In the absence of correction, performance was markedly reduced (cross-validation mean AUC: 0.51 ± 0.28; institute holdout mean AUC: 0.64 ± 0.20). After correction, performance improved substantially (10-fold cross-validation mean AUC: 0.99 ± 0.05; institute holdout mean AUC: 0.89 ± 0.12).

Given the enrichment of telomeric and keratinization-associated loci in differential rMDS clusters, we also evaluated whether restricting features to these focal regions retained predictive value (Supplemental Figure 17). Models trained using rMDS derived exclusively from keratinization loci showed moderate discrimination (mean AUC = 0.67 ± 0.21), whereas telomere-proximal regions demonstrated improved performance (mean AUC = 0.76 ± 0.28). Combining keratinization and telomeric loci maintained comparable performance (mean AUC = 0.73 ± 0.32). These results suggest that specific biologically enriched regions harbor concentrated fragmentation signals that may serve as feature subsets for future biomarker development with increased coverage.

### rMDS-based response predictions correlate with survival outcomes and enhance risk stratification.

**Figure 5:**
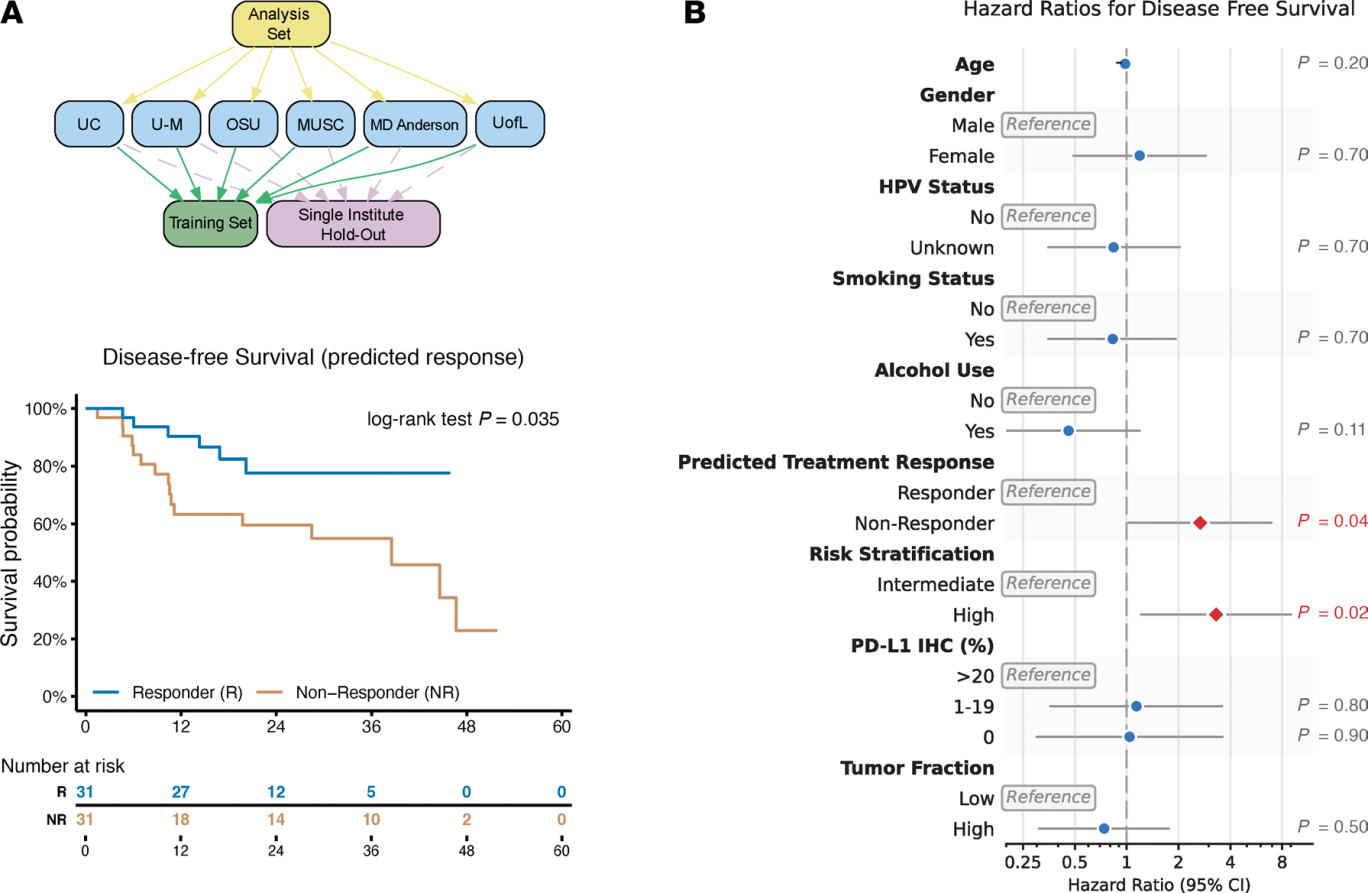
cfDNA rMDS-based model predicts clinical outcomes in patients with HNSCC treated with pembrolizumab. (**A**) DFS stratified by predicted treatment response (responder vs. nonresponder) using a cfDNA rMDS-based model with a single-institute holdout evaluation strategy. The global log-rank *P* value and risk table are shown. (**B**) HRs for DFS across clinical and demographic covariates, estimated by Cox proportional hazards regression (*P* values indicated).

We next evaluated whether rMDS-based response predictions could serve as prognostic biomarkers. Five-year DFS and overall survival (OS) were analyzed according to the predicted response status using the single-institute holdout evaluation. Survival analyses were based on the predicted response status from the single-institute holdout evaluation, restricted to patients with available adjuvant week 1 samples. Notably, despite the single-institute holdout achieving 74.2% accuracy, patients predicted as responders exhibited significantly improved DFS compared with predicted nonresponders (log-rank test, *P* = 0.035; Figure 5A). This prognostic capability was superior to established biomarkers, as no significant differences were observed when using the PD-L1 combined positive score (CPS) (*P* = 0.97) or tumor fraction (*P* = 0.50) from the same patients. The rMDS predictions demonstrated a prognostic value comparable to that of pathological risk stratification (*P* = 0.013; Supplemental Figure 18).

For OS, although rMDS-predicted responders showed a trend toward improved outcomes (log-rank test, *P* = 0.197), this did not reach statistical significance. In comparison, neither the PD-L1 CPS (*P* = 0.52) nor the tumor fraction (*P* = 0.15) showed prognostic value for OS, whereas pathological risk remained significant (*P* = 0.002, Supplemental Figure 19, A–D). HR analysis revealed that rMDS-predicted nonresponders had significant HRs of 2.67 (95% CI: 1.03–6.92) for DFS, demonstrating the clinical utility of this biomarker approach. While no statistically significant HR was observed for OS with rMDS predictions (1.98, 95% CI: 0.69–5.74), pathological risk showed significant HRs for both DFS (3.31, 95% CI: 1.21–9.01) and OS (7.16, 95% CI: 1.62–31.6) (Figure 5B, Supplemental Figure 19E, and Supplemental Table 7). Since some patients may have experienced recurrence between neoadjuvant and adjuvant treatments, we further adjusted the survival time to reflect the months since neoadjuvant pembrolizumab initiation, and the results remained similar. (Supplemental Figures 20 and 21, and Supplemental Table 7).

These findings suggest that rMDS-based predictions may provide clinically meaningful risk stratification and short-term outcome prediction, outperforming conventional markers like PD-L1 CPS and tumor fraction and offering the potential to enhance future clinical risk stratification models.

## Discussion

This study represents, to our knowledge, the largest prospective, multi-institutional investigation of cfDNA fragmentation in HNSCC to date. We demonstrate that the diversity of cfDNA end motifs, quantified by the rMDS, can distinguish immunotherapy responders from nonresponders in an unsupervised manner. Utilizing low-coverage WGS data from a cohort treated with neoadjuvant pembrolizumab, we show that rMDS separated responders from nonresponders with high accuracy and correlated with DFS more robustly than conventional biomarkers, such as PD-L1 expression or tumor fraction.

Our study provides several insights. First, rMDS, derived from the entropy of 5′ end motifs in 500 kb bins, captured the longitudinal dynamics of region-specific fragmentation changes in an unsupervised manner, outperforming other fragmentomic features and genetic aberrations. Unlike previous fragmentomic approaches that summarized end-motif diversity across the entire genome, rMDS quantified regional variation, capturing biologically localized chromatin differences associated with immunotherapy response. Remarkably, rMDS was able to distinguish responders from nonresponders even at the pretreatment screening time point, suggesting that baseline cellular epigenetic plasticity may predict future responsiveness to immunotherapy. This observation warrants further experimental validation using matched tumor and immune cell samples. While prior studies have shown the utility of genome-wide MDSs in distinguishing patients with HNSCC from healthy individuals (18), our results highlight the limitations of relying on a single summary statistic for predicting treatment response. The original MDS compresses the diversity of 5′ end motifs into a single value and ignores the baseline variation across individuals, potentially masking important regional fragmentation dynamics.

Hierarchical clustering of differentially fragmented regions revealed distinct patterns of divergence between responders and nonresponders, with region-specific enrichments originating from both tumor and immune compartments, suggesting that tumor-immune interactions shape patients’ responses to immunotherapy. Clusters 2 and 3 comprised loci with persistent baseline differences and were highly enriched for immune, CENPA, and lectin signaling–related genes, which have previously implicated immunotherapy responses in other cancer types and chemotherapy treatment outcomes, as well as in HNSCC disease progression and prognosis. In contrast, cluster 1 contained regions exhibiting more dynamic changes following treatment and was enriched for keratinization-related genes — the histologic hallmark of squamous cell carcinoma — highlighting the potential role of tumor-derived cfDNA or alterations in tumor cell epigenomes during immunotherapy. Interestingly, cluster 1 regions were preferentially localized near telomeric ends, which are highly associated with genomic instability (38) and may reflect different cell death mechanisms, such as apoptosis and necrosis. Emerging evidence also suggests that telomere maintenance genes influence immune cell infiltration and response to immunotherapy (39, 40). However, the relationship between telomere biology and cfDNA fragmentation, particularly regarding end-motif diversity, remains unexplored and needs further investigation.

Our study also has several limitations. The relatively low sequencing coverage (~1.9×) and patient heterogeneity limited our ability to investigate rMDS at focal gene-regulatory regions, such as transcription start sites (TSSs) and transcription factor binding sites in individual patients. Although our results suggest a potentially distinct fragmentation program near telomeric regions during immunotherapy — potentially driven by chromatin organization, genomic instability, or differences in cell death mechanisms between patient groups — the limited sequencing depth precluded higher-resolution exploration of rMDS in these regions. However, our empirical downsampling analysis demonstrates that the biological signal captured by rMDS was robust and not exclusively dependent on high-depth sequencing. While deeper sequencing may enhance precision and reduce variance at critical gene-regulatory elements, the discriminatory structure underlying rMDS remains robust at the coverage levels utilized in this study. Similarly, although cluster 1 was enriched for keratin-associated loci, the low coverage and lack of complementary gene expression or methylation data in matched tissues prevented definitive attribution of observed changes to epithelial cell–specific processes. Furthermore, the low sequencing depth (~1.9×) precluded reliable somatic mutation calling, which is a prerequisite for personalized, tumor-informed ctDNA assays such as Signatera. Mutation-based approaches, by virtue of tracking tumor-specific somatic variants, offer high specificity and are well suited to clinical settings where matched tumor tissue and high-depth sequencing are available. While mutation-based ctDNA has demonstrated strong prognostic value for recurrence detection in HNSCC (41, 42), its application to the specific context of predicting immunotherapy responses has not been systematically evaluated. In contrast, our rMDS-based approach derives signal from cfDNA fragmentation patterns rather than somatic mutations and does not require matched tumor tissue or high sequencing depth, making it particularly applicable to low-coverage sequencing settings and potentially well suited to capturing immunotherapy-related biological signals. The HR observed with rMDS-predicted responses for DFS (HR 2.67; 95% CI 1.03–6.92) was directionally consistent with, though more modest than, the HRs reported by mutation-based assays in the context of recurrence surveillance. Future studies integrating rMDS with tumor-informed, mutation-based ctDNA detection may further enhance prognostic stratification in this disease setting.

Our analysis also faced challenges related to pronounced batch effects. To address this, we trained a batch correction model using the plasma collection site, the cfDNA isolation date, and the WGS sequencing date as covariates, while explicitly preserving the component associated with treatment response. This approach was designed to remove technical variation while maintaining the rMDS signal’s discriminatory power. Although the batch correction model was fit on the full analysis set, including all institutes, it is important to note that the pre-holdout set was not included in model fitting. As a result, evaluation in the pre-holdout set remains valid, albeit with lower performance. Importantly, this analysis was included to demonstrate the effect of incomplete batch correction on fragmentomic predictors rather than to serve as the primary measure of model performance. Future work should focus on developing improved batch correction strategies specifically tailored for cfDNA fragmentation data. Additionally, further validation in independent cohorts, ideally with deeper cfDNA WGS coverage, is a critical next step.

Moreover, our reliance on engineered features, such as the arbitrary 4 mer motif length at the 5′ end, while interpretable, may underutilize the rich sequence context present in cfDNA fragments. Recent studies have suggested that transformer-based models, which capture long-range dependencies and positional information in DNA, could be valuable in this context. Future work should explore the use of attention-based architectures trained directly on cfDNA sequences, as these may reveal informative embeddings beyond motif frequency or entropy, especially in low-depth sequencing scenarios.

Finally, although our results were validated across holdout splits and multiple time points, validation in external cohorts with alternative therapeutic regimens will be necessary to fully elucidate patient response mechanisms to immunotherapy. All patients in this study received neoadjuvant pembrolizumab and underwent surgery, which may limit generalizability to recurrent/metastatic cases and other clinical settings. Prospective validation in independent cohorts, particularly those treated with different immunotherapy regimens, will be valuable. We also note that the prognostic utility of rMDS appears primarily suited for short-term response prediction rather than long-term survival. Although rMDS served as a robust indicator of DFS, its association with OS did not reach statistical significance, warranting a more moderated interpretation of its broader prognostic applicability. This distinction may partly reflect the current follow-up duration as well as the potential confounding effects of subsequent lines of therapy on OS. Importantly, early-event endpoints are increasingly recognized as clinically meaningful measures of therapeutic benefit. For example, the recent FDA approval of neoadjuvant and adjuvant pembrolizumab for resectable locally advanced HNSCC (KEYNOTE-689) (20) was based on improvements in event-free survival — a composite endpoint encompassing disease recurrence, progression preventing definitive surgery, or death — rather than OS. In a similar conceptual framework, DFS served as the primary endpoint in our study and represents an analogous early measure of treatment outcome. Consequently, while the rMDS shows promise for early risk stratification based on DFS, further validation in larger cohorts with extended follow-up will be necessary to determine its definitive role in predicting long-term survival outcomes.

In conclusion, this study establishes the cfDNA rMDS as a biologically meaningful and clinically predictive biomarker for immunotherapy response in HNSCC. By introducing the rMDS as a temporally dynamic signal, we provide a framework for stratifying patients on the basis of treatment response, with the potential to inform and personalize clinical decision-making. Future studies are warranted to evaluate the rMDS in broader cancer types, to integrate it with other multimodal biomarkers, and to assess its clinical utility in real-time therapeutic settings.

## Methods

### Sex as a biological variable.

Our study examined male and female individuals, and there was no significant effect of sex on the conclusion.

### Study cohort and sample collection.

This prospective, multi-institutional phase II clinical trial (NCT02641093) enrolled patients with newly diagnosed, surgically resectable, locally advanced HNSCC receiving neoadjuvant and adjuvant immunotherapy.

Patients were recruited from 6 academic centers: UC, University of Michigan (U-M), Ohio State University (OSU), Medical University of South Carolina (MUSC), MD Anderson Cancer Center (MDACC), and University of Louisville (UofL). Each patient received a single i.v. dose of pembrolizumab (200 mg) 7–21 days prior to surgery, followed by adjuvant radiotherapy (60–66 Gy in 30–33 fractions). High-risk patients received concurrent weekly cisplatin (40 mg/m^2^), and all patients were eligible for adjuvant pembrolizumab (200 mg i.v. every 3 weeks, up to 6 doses).

Eligibility criteria included age 18 years or older, newly diagnosed and histologically or cytologically confirmed HNSCC, and locally advanced disease classified as stage III or IV according to the *American Joint Committee on Cancer (AJCC) 8th Edition* (T3 or T4 tumor, ≥N2 nodal disease, or clinical evidence of extranodal extension [ENE] on imaging). Tumors had to be deemed resectable by the treating head and neck surgeon, with no involvement of the skull base or T4b stage. Additional inclusion criteria were an Eastern Cooperative Oncology Group (ECOG) performance status of 1 or less and adequate organ function.

Key exclusion criteria were human HPV^+^ oropharyngeal cancer (HPV^+^ disease outside the oropharynx was permissible, although HPV testing was not mandatory), nasopharyngeal cancer, metastatic disease (as determined by chest CT or PET/CT), autoimmune disease, active intercurrent illness (including significant cardiovascular disease, active viral infections, or major psychiatric illness), and steroid use exceeding prednisone 10 mg daily. For comprehensive details, refer to the original study (19).

Peripheral blood collected at the first 3 time points was used in this study: (a) pretreatment screening (screen); (b) immediately after surgical resection (day 0); and (c) approximately 3–10 weeks following surgery (adjuvant week 1). We included all plasma samples available from the trial for cfDNA analysis. Samples for which only whole blood was collected were excluded, as genomic DNA contamination in whole-blood specimens can compromise the integrity of cfDNA fragmentation measurements.

### Treatment response and pathological risk determination.

The pathological response to neoadjuvant pembrolizumab was evaluated by centralized histopathological analysis. Resected tumor specimens were assessed by experienced pathologists for standard pathological risk features, including extracapsular nodal extension (ENE), margin status, lymphovascular invasion, perineural invasion, and the number of involved lymph nodes. For comprehensive details, refer to the original study (19).

The primary metric for treatment response was the pathological TE, defined as tumor necrosis accompanied by histiocytic inflammation and/or giant cell reaction to keratinaceous debris. TE was quantified as the ratio of the area showing these features to the total area comprising residual viable tumor and the TE. Pathological response was classified as no response (TE <10%) or response (TE ≥10%), similar to previous studies (20, 43). The presence of positive surgical margins and/or ENE was used to define pathological risk. For comprehensive details, refer to the original study (19).

### Plasma processing and cfDNA extraction.

Peripheral blood was shipped overnight, and cfDNA was isolated from 500 μL plasma, with all samples stored at –80°C prior to processing. Before isolation, plasma samples were thawed on ice, centrifuged at 1,600*g* for 10 minutes at 4°C, and then subjected to a second centrifugation at 16,000*g* for 10 minutes at 4°C to remove residual cell debris. cfDNA extraction was performed using the MagMAX Cell-Free DNA Isolation Kit (Applied Biosystems) following the manufacturer’s protocol. cfDNA concentration and size distribution were measured using Qubit (Invitrogen, Thermo Fisher Scientific) and BioAnalyzer (Agilent Technologies), respectively. For each patient, plasma samples collected at 3 different time points were processed together within the same isolation batch.

### cfDNA WGS.

cfDNA samples were randomly allocated into 8 library preparation batches, ensuring that all time points from each patient were included in the same batch. Library preparation was performed on 1 ng cfDNA using the KAPA HyperPrep Kit (Roche) and NEXTFLEX Unique Dual Index Barcodes (PerkinElmer; 300 nM final concentration). Sequencing was carried out on the Illumina NovaSeq 6000 S4 platform using paired-end 150 bp reads (PE150).

### Data preprocessing of cfDNA WGS data.

Raw sequencing data were processed using the FinaleDB Workflow (44) (https://github.com/epifluidlab/finaledb_workflow), implemented via Snakemake (version 7.8.0) (45). Specifically, paired-end FASTQ files underwent adapter trimming with Trimmomatic (version 0.39) (46), followed by alignment to the human reference genome (hg38) using BWA-MEM (version 0.7.15) (47) with default parameters. PCR duplicates were removed with samblaster (version 0.1.24) (48). Reads were retained if both ends were uniquely mapped, properly paired, nonsupplemental, and had a mapping quality of 30 or greater. Only autosomal fragments of 50–350 bp in length were included. Reads overlapping ENCODE blacklist regions (49) (hg38) were excluded to eliminate mapping artifacts.

### rMDS.

The autosome genome was divided into 500 kb nonoverlapped bins. For each 500 kb bin *j*, the raw rMDS was defined as the normalized Shannon entropy of 5′ end 4 mer motifs:
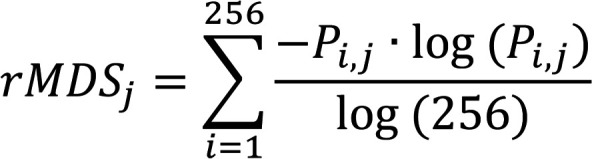


where *P_i,j_* is the relative frequency of the *i_th_* 4 mer end motif in bin *j*. Normalization by log(256) ensures that the score ranges from 0 (minimum diversity) to 1 (maximum diversity). The resulting rMDS values were then *z* score transformed across all bins in each sample,
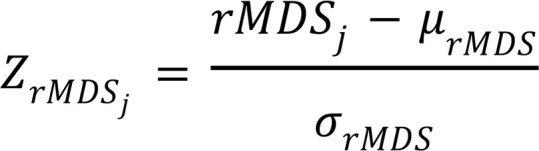


where *μ_rMDS_* and *σ_rMDS_* denote the mean and SD of rMDS values across the genome, respectively. These *z*-transformed values were used in all downstream analyses. The 500 kb bin size provided a balance between genomic resolution and fragment count per bin (~2 × 10^4^ fragments/bin at 1.9× coverage).

### Other cfDNA fragmentation feature extraction.

Additional fragmentation features were extracted from preprocessed BED and BAM files using FinaleToolkit (version 0.10.7) (21). The autosomal genome was divided into nonoverlapping 500 kb bins using Bedtools (version 2.31.1) (50), excluding low-mappability regions identified by the UCSC 45 mer mappability track (hg38) (51). Fragment-level BED files were used to calculate: (a) fragment length distributions, (b) 4 mer fragment end-motif frequencies, (c) global MDSs, and (d) DELFI scores (with the --no merge-bins option). GC-corrected coverage values were derived from DELFI-corrected fragment counts. For copy number and tumor fraction analyses, BAM files were processed using ichorCNA (version 0.2.0) (22) with hg38 genome settings at the same resolution.

### Batch correction and dimensionality reduction.

Batch correction of rMDS values and other fragmentomic features was performed using a linear model adapted from the limma framework (52). For each 500 kb genomic bin, rMDS values were modeled as a function of cfDNA isolation date, WGS library preparation date, and collection institute, with treatment response (responder vs. nonresponder) included as a preserved biological covariate. Design matrices employed sum contrasts via the patsy.contrasts.sum method (patsy version 0.5.6). Linear coefficients were estimated on the analysis set (including the Institute Holdout set) using ordinary least squares, and batch effects were regressed out. To preserve the independence of the pre-holdout set, batch correction was performed by applying the design matrix contrasts and regression coefficients learned from the analysis set. No parameters were re-estimated using pre-holdout data. Matrix operations were implemented with NumPy (version 1.26.4) (53) and pandas (version 2.2.2) (54).

Corrected matrices were standardized using *z* score normalization (StandardScaler, scikit-learn version 1.5.2) (55). Dimensionality reduction was conducted using truncated SVD (numpy.linalg.svd) (53), with components selected according to the singular value spectrum and variance explained (Supplemental Figure 5).

Low-dimensional embeddings were generated using UMAP (umap-learn version 0.5.7; metric=’cosine’, n_neighbors=50, min_dist=0.5) (56). Confidence ellipses (2 SD) for each group were constructed from the eigen decomposition of the covariance matrix, and ellipse intersections were computed using Shapely (version 2.1.0). Visualizations were produced with Matplotlib (version 3.8.3) (57) and Seaborn (version 0.13.2) (58). To quantify the quality and stability of the resulting clusters, specifically the separation between responders and nonresponders, we calculated Silhouette scores. The Silhouette score for each sample was computed as (*b − a*)/max(*a,b*)*,* where *a* is the mean intracluster distance and *b* is the mean distance to the nearest neighboring cluster.

### Sequencing depth and stability analysis.

To evaluate the effect of sequencing depth on rMDS, we used a reference cfDNA WGS sample from BH01 in Snyder et al. (13) (56× effective coverage) and performed empirical downsampling to 50×, 25×, 10×, 5×, 2×, 1×, and 0.1× effective coverage. Pearson correlation coefficients were computed between each downsampled profile and the 56× reference to assess signal recovery. Additionally, responder and nonresponder samples were downsampled to assess the stability of group-level biological discrimination across varying depths.

### Differential analysis of rMDS.

Differential analysis of rMDS between treatment responders and nonresponders was performed using the limma (52) package in R with a linear mixed-effects modeling approach to account for repeated measures across time points. A global design matrix incorporating treatment response group and clinical covariates (age, race, sex, ethnicity, diagnosis, smoking, alcohol use) was constructed, with within-patient correlation estimated using patient ID as a blocking variable and array-specific weights applied to handle variance heterogeneity. Linear models were fitted using empirical Bayes moderation, and raw *P* values were corrected for multiple testing using *q* values to control the FDR. Genomic bins with *q* values of less than 0.1 were considered significantly differential between responders and nonresponders.

### Clustering and chromosomal enrichment of differential rMDS regions.

Using clinical covariate-corrected (Supplemental Figure 9) and uncorrected rMDS data (Figure 3), differential 500 kb bins were clustered based on rMDS values across the 3 time points (screen, day 0, adjuvant week 1). Genomic bins (columns) were hierarchically clustered using Ward’s method (scipy.cluster.hierarchy.linkage) (59), with clusters determined via fcluster (t=3, criterion=’maxclust’). Patient rows were independently clustered within each treatment group. Heatmaps were generated with Marsilea (version 0.5.1) (60).

For each cluster, the average rMDS trajectory across time points was computed and stratified by response group. Longitudinal patterns were visualized as line plots (Seaborn, version 0.13.2, ci=95) (58).

Telomeric enrichment was assessed by calculating the distance from each bin midpoint to the nearest chromosome end using hg38.chrom.sizes (UCSC) (51). For each cluster, the empirical distance distribution was compared with a null distribution generated from 100,000 randomly sampled 500 kb bins, using a 1-sided Mann-Whitney *U* test (scipy.stats.mannwhitneyu) (59) and a permutation test to determine whether observed distances were significantly shorter than expected. Density plots were generated with kernel density estimation (scipy.stats.gaussian_kde) (59), normalized for comparison.

### Gene ontology and pathway enrichment analysis of differential rMDS regions.

Functional enrichment analysis was performed for each rMDS cluster using g:Profiler (version e112_eg59_p19_25aa4782) (61). Genomic bins were mapped to nearby genes using default Ensembl annotations. Enrichment of GO Biological Process and Reactome pathway terms was tested, and fold enrichment was calculated as (intersection size/query size) divided by (term size/effective domain size). The top 10 significant enriched terms with the highest fold changes were visualized in the main figure.

### Locus-specific visualization of differential rMDS signal profiles.

Genome-wide sliding windows of 500 kb (step size: 10 kb) were generated. Basically, fragment end-motif profiles were computed using the interval-end-motifs and rmds modules from FinaleToolkit (version 0.10.7) (21) in each 10 kb window using the rMDS value within the nearby 500 kb window (± 250 kb from the center of 10 kb window). Signal matrices were *z* score normalized, batch corrected, and averaged by group (response and time point). Visualization was performed with IGV (version 2.19.2) (62), using color-coded overlays to differentiate response groups and time points.

### Machine learning classification of the treatment response.

To evaluate the predictive power of rMDS features, models were trained using TabPFNClassifier (tabpfn version 2.0.8) (37). Input matrices were batch-corrected, *z* score normalized, and reduced via truncated SVD (scikit-learn version 1.5.2, n_components=6) (55). Model performance was assessed using ROC AUC, accuracy, F1 score, precision, recall, and balanced accuracy. Patient-level predictions were obtained using the most recent available time point (adjuvant week 1 or day 0).

Model robustness was tested to evaluate the effect of random sampling by 3 approaches. (a) Cross-validation evaluation: The model underwent 100 rounds of 10-fold cross-validation at the patient level. In each round, patients were randomly divided into 10 groups, with 9 groups used for training and 1 group held out for testing. This process was repeated 10 times per round (rotating which group was held out) and then replicated 100 times with different random splits. (b) Single-institute holdout evaluation: The model was trained using data from 5 institutes, and then tested separately on data from the that was held out. (c) Pre-holdout evaluation: The model underwent 100 rounds of testing, in which 10 patients were randomly selected as the test set in each round. Importantly, any batch correction factors were learned only from the training set, ensuring the test remained truly independent.

Performance metrics were computed at both the sample and patient levels. ROC curves and confusion matrices were visualized using Seaborn (version 0.13.2) (56) and Matplotlib (version 3.8.3) (57).

### Survival analysis.

Survival analyses were performed in R (version 4.5.0) using survival (version 3.8.3) (63, 64). Kaplan-Meier estimates for DFS and OS were calculated, with time-to-event defined as days from the adjuvant week 1 dose. Patients missing this time point were excluded from downstream analysis.

PD-L1 expression was assessed using IHC. Formalin-fixed, paraffin-embedded tumor tissue samples were stained using the 22C3 antibody clone (Agilent Technologies, Dako) and evaluated with the FDA-approved PD-L1 IHC pharmDx assay. Testing was performed at Caris Life Sciences or NeoGenomics Laboratories and independently confirmed by a board-certified pathologist at the University of Cincinnati.

The CPS was calculated as follows: CPS = (number of PD-L1^+^ tumor cells + PD-L1^+^ immune cells) × 100/(total number of viable tumor cells). For comprehensive details, refer to the original study (19).

Survival analyses were stratified by the predicted treatment response (responder vs. nonresponder) from the single-institute holdout evaluation, pathological risk categories (intermediate vs. high), PD-L1 CPS categories (>20, 1–19, 0), and tumor fraction (low vs. high, dichotomized at the median). Kaplan-Meier survival curves were generated with accompanying risk tables showing the number of patients at risk. For group comparisons, log-rank tests were applied (63). Visualizations were created using ggplot2 (version 3.5.2) (65), with pairwise comparisons performed for multilevel groups.

Cox proportional hazards regression models were fitted to estimate HRs with 95% CIs. Univariate analyses were performed for age, sex, HPV status (determined by p16 IHC as a surrogate), smoking history, alcohol use, predicted response, risk stratification, PD-L1 expression, and tumor fraction. Results were considered statistically significant at a *P* value of less than 0.05.

### Statistics.

Cluster separation between immunotherapy responders and nonresponders was quantified using silhouette scores. Pearson correlation coefficients were used to assess agreement between downsampled and full-depth rMDS profiles. Telomeric enrichment of differential rMDS clusters was evaluated using 1-sided Mann-Whitney *U* and permutation tests. Differential rMDS analysis between responders and nonresponders was performed using linear mixed-effects models within the limma framework, with empirical Bayes moderation; multiple testing correction was applied using *q* values, with a FDR threshold of a *q* < 0.1. Kaplan-Meier survival curves were compared using log-rank tests, and HRs with 95% CIs were estimated using Cox proportional hazards regression. All other tests were 2 sided, and results were considered statistically significant at a *P* value of less than 0.05. Unless otherwise indicated (e.g., Figure 2B ellipses represent approximately 2 SDs from group centroids), data in figures are presented as mean ± SD. ROC curves and confusion matrices show mean ± SD across validation iterations; hazard ratios are shown with 95% confidence intervals. Kaplan-Meier estimates are shown with number-at-risk tables; log-rank P values are indicated.

### Study approval.

The study was approved by the IRBs of all participating sites: University of Cincinnati, Cincinnati, Ohio, USA; University of Michigan, Ann Arbor, Michigan, USA; The Ohio State University, Columbus, Ohio, USA; Medical University of South Carolina, Charleston, South Carolina, USA; The University of Texas MD Anderson Cancer Center, Houston, Texas, USA; and University of Louisville, Louisville, Kentucky, USA. The study was conducted in accordance with the Declaration of Helsinki and Good Clinical Practice guidelines. Written informed consent was obtained from all participants prior to study procedures.

### Data availability.

All analysis code is available at the study’s GitHub repository (https://github.com/epifluidlab/headneck). Supporting data values for all figures, including data points shown in graphs and values underlying reported means, are provided in the Supporting Data Values file. Raw WGS data from plasma cfDNA will be available in the European Genome-phenome Archive (EGA) with controlled access (accession code EGAS50000001286. Data access can be obtained through a request to the corresponding authors. The corresponding authors will generally respond to requests within 1 week. Once granted, the access has no time restriction. The raw sequencing data are protected by data privacy laws. The deidentified, post-processed fragment files are available in zenodo.org (doi: 10.5281/zenodo.15738112).

## Author contributions

YL and TWD conceived the study. HF and LW performed the cfDNA extraction and library constructions. RB, HZ, and JL performed the data analysis with input from YL, TWD, SG, MT, BZ, MK, DL, and DAH. FPW, MOO, NED, JMK, MG, DEG, BHH, and TWD designed the clinical trial and provided the blood samples. RB and YL wrote the manuscript together. All authors read and approved the final manuscript.

## Conflict of interest

YL owns stocks in Freenome Inc. TWD received research funding to conduct this clinical trial from Merck Sharp & Dohme Corp., a subsidiary of Merck & Co. Inc.

## Funding support

This work was supported in part by funding from the National Institutes of Health. In accordance with the NIH Public Access Policy, the NIH has been given a right to make the work publicly available in PubMed Central.

- Cincinnati Children’s Hospital Medical Center, Northwestern University, and the Robert H. Lurie Comprehensive Cancer Center of Northwestern University (startup grant, to YL).
- National Human Genome Research Institute (grant R56HG012360, to YL).
- Science Olympiad Alumni Research (SOAR) Grant, Science Olympiad USA Foundation (to RB).
- Merck Sharp & Dohme Corp., a subsidiary of Merck & Co. Inc. (funding for the conduct of this study, to TWD).
- Computational resources and staff contributions provided for the Quest high performance computing facility at Northwestern University, which is jointly supported by the Office of the Provost, the Office for Research, and Northwestern University Information Technology.
- Computational resources and staff contributions provided by the Genomics Compute Cluster, which is jointly supported by the Feinberg School of Medicine, the Center for Genetic Medicine, and Feinberg’s Department of Biochemistry and Molecular Genetics, the Office of the Provost, the Office for Research, and Northwestern Information Technology.

## Supplementary Material

Supplemental data

ICMJE disclosure forms

Supplemental tables 1-7

Supporting data values

## Figures and Tables

**Table 1:**
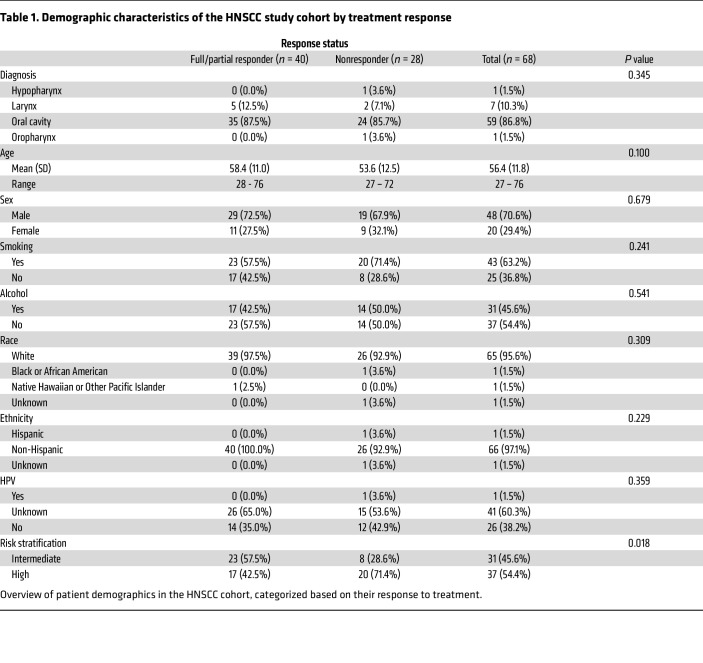
Demographic characteristics of the HNSCC study cohort by treatment response

## Version 1 05/19/2026

In-Press Preview

## Version 2 07/01/2026

Electronic publication
